# The Reflective Teaching Log (RTL): Effective Documentation of Participatory Teaching Requirements

**DOI:** 10.1007/s40670-019-00709-1

**Published:** 2019-03-05

**Authors:** Elisabeth F. M. Schlegel, Michael Cassara, Doreen M. Olvet, Alice Fornari

**Affiliations:** Donald and Barbara Zucker School of Medicine at Hofstra/Northwell, Hempstead, NY USA

**Keywords:** Teaching log, Reflection, Medical education elective, Course requirement

## Abstract

We created a reflective teaching log for a student-as-teacher elective to track students’ required participatory teaching and to provide a mechanism for reflective evaluation. This Minute Paper–based log is easy to use and can be adapted to similar programs capturing insights of students’ teaching experiences and supports reflective learning.

The professional development of medical students has expanded to include a role as an educator. As future physicians, they will be expected to teach medical students and residents both during residency training and as faculty physicians in a university hospital setting. Recognizing this, at least 44% of medical schools now offer medical-student-as-teacher (MST) programs [1]. These programs traditionally include peer or near-peer teaching as well as didactic sessions on the knowledge and skills that are essential to become effective teachers [2]. However, MST students do not necessarily receive feedback from faculty on their teaching performance [2]. A mechanism should be in place to record teaching experiences and allow for an exchange of reflective feedback with the course director about each teaching encounter.

The practice of reflective writing can promote learning through transformation of experience into abstract conceptualization and insights followed by application [3]. Reflective logs have been used to document student experiences during clinical teaching and learning sessions [4]. Data from these student reflections indicate that medical students develop teaching skills, self-confidence, and a professional identity as a medical educator [4]. However, these findings are limited because it may not be practical to gather lengthy reflections after all pivotal teaching experiences [4]. One solution is to incorporate brief Minute Paper elements within a participatory teaching requirement log [5]. The Minute Paper is an assessment technique that allows learners to briefly reflect on and assess their understanding of the material [5].

We developed a brief Reflective Teaching Log (RTL) as a part of our MST elective course offered in the fourth year at the Zucker School of Medicine at Hofstra/Northwell. The MST course is longitudinal and consists of a combination of didactic lectures, workshops, journal club sessions, and a summative capstone project. In addition, students are required to complete 20 h of self-selected teaching, which is documented in the RTL. The RTL was created in Microsoft Excel and is made available to students electronically and stored in a cloud-based platform (e.g., Google docs). Each student shares a link with the Course Directors. This shared spreadsheet streamlined the communication process between students and faculty by allowing for real-time data sharing on their teaching experiences. Students are required to complete the log for each teaching experience including logistical information, educational setting they taught, pedagogy employed, the number of hours taught, their role, and their faculty supervisor. In addition, the major portion of the RTL is comprised of elements of the Minute Paper, such as “most important concept learned,” or “concept requiring clarification” (see Fig. 1, simulated Reflective Teaching Log RTL). These columns prompt students to briefly reflect on their experience more broadly, asking them to describe any insights gained, the most important concept they learned, what they needed clarification on, and what specific skill they will take away from the experience. The logs also provided important program related feedback, such as which sessions or pedagogies were most valuable to learners and the diversity of teaching sessions selected.

**Fig. 1:**
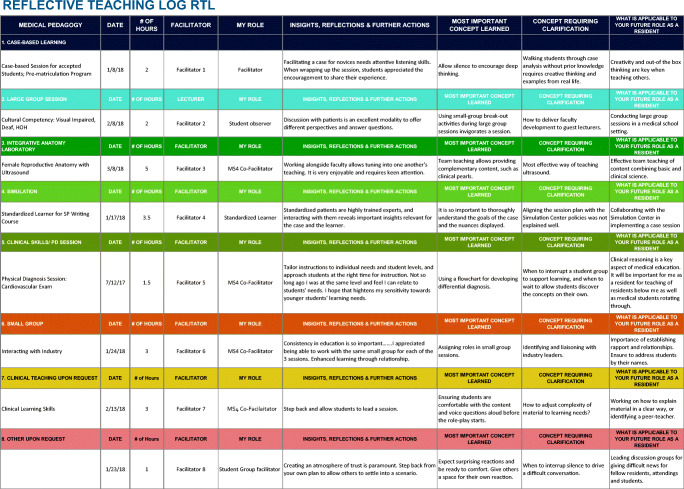
Reflective Teaching Log RTL

The RTL is an ideal tool for tracking student teaching and reflective experiences without being overly burdensome for the student or Course Director. It can easily be adapted to different educational settings, and individual variables can be tailored to specific course objectives. We encourage other medical schools to utilize the RTL to track program outcomes quantitatively and qualitatively. The outcomes will ultimately improve curricula focused on training MSTs.

## References

[1] Soriano RP, Blatt B, Coplit L, Cichoski Kelly E, Kosowicz L, Newman L, Pasquale SJ, Pretorius R, Rosen JM, Saks NS, Greenberg L (2010). Teaching medical students how to teach: a national survey of students-as-teachers programs in US medical schools. Acad Med.

[2] Marton GE, McCullough B, Ramnanan CJ (2015). A review of teaching skills development programmes for medical students. Med Educ.

[3] Kolb DA. Experiential learning: experience as the source of learning and development: FT press; 2014.

[4] Freeman M (2001). Reflective logs: an aid to clinical teaching and learning. Int J Lang Commun Disord.

[5] Angelo TA, Cross KP (1993). Classroom assessment techniques: a handbook for faculty.

